# Intermittent Courses of Corticosteroids Also Present a Risk for* Pneumocystis* Pneumonia in Non-HIV Patients

**DOI:** 10.1155/2016/2464791

**Published:** 2016-09-18

**Authors:** Maria L. Calero-Bernal, Isabel Martin-Garrido, Mikel Donazar-Ezcurra, Andrew H. Limper, Eva M. Carmona

**Affiliations:** ^1^Division of Pulmonary and Critical Care, Department of Medicine, Mayo Clinic, Rochester, MN, USA; ^2^Servicio de Medicina Interna, Hospital Universitario Virgen del Rocío, Seville, Spain; ^3^Servicio de Medicina Interna, Hospital Quirón San Camilo, Madrid, Spain; ^4^Complejo Hospitalario de Navarra, Pamplona, Navarra, Spain

## Abstract

*Introduction*.* Pneumocystis* pneumonia (PCP) is rising in the non-HIV population and associates with higher morbidity and mortality. The aggressive immunosuppressive regimens, as well as the lack of stablished guidelines for chemoprophylaxis, are likely contributors to this increased incidence. Herein, we have explored the underlying conditions, immunosuppressive therapies, and clinical outcomes of PCP in HIV-negative patients.* Methods*. Retrospective analysis of PCP in HIV-negative patients at Mayo Clinic from 2006–2010. The underlying condition, immunosuppressive therapies, coinfection, and clinical course were determined. PCP diagnosis required symptoms of pneumonia and identification of the organisms by visualization or by a real-time polymerase chain reaction.* Results*. A total of 128 cases of PCP were identified during the study period. Hematological malignancies were the predisposing condition for 50% of the patients. While 87% had received corticosteroids or other immunosuppressive therapies for >4 weeks prior to the diagnosis, only 7 were receiving PCP prophylaxis. Up to 43% of patients were not on daily steroids. Sixty-seven patients needed Intensive Care Unit (ICU) and 53 received mechanical ventilation. The mortality for those patients requiring ICU was 40%.* Conclusions*. PCP diagnosis in the HIV-negative population requires a high level of suspicion even if patients are not receiving daily corticosteroids. Mortality remains high despite adequate treatment.

## 1. Introduction

*Pneumocystis jirovecii* is an opportunistic pathogen that causes pneumonia in immunocompromised patients. It carries significant mortality in patients with Human Immunodeficiency Virus (HIV) infection and is considered a principal acquired immunodeficiency syndrome- (AIDS-) defining illness. Since the widespread use of prophylaxis and the initiation of highly active antiretroviral therapy, the number of cases of* Pneumocystis* pneumonia (PCP) during HIV infection has decreased significantly. In contrast, PCP is growing among HIV-negative patients [1–3]. The use of diverse immunosuppressive agents and corticosteroids for treatment of various underlying conditions (hematological malignancies, rheumatologic processes, and bone marrow or solid organ transplant) is resulting in increasing numbers of PCP in HIV-negative patients [4].

PCP in settings other than AIDS usually manifests in a more fulminant manner [5]. Studies of bronchoalveolar lavage (BAL) obtained during PCP demonstrated considerably fewer organisms in the lungs of HIV-negative patients than in those with HIV infection. In contrast, HIV-negative patients exhibited a more exuberant lung inflammation and more profound hypoxemia [6]. Furthermore, the overall mortality attributable to PCP during AIDS ranges from 10 to 20%, whereas the mortality in HIV-negative patients remains approximately 30–50% and has remained unchanged over the last two decades [7].

Classically, corticosteroids and other cytotoxic agents such as methotrexate, cyclosporine, and cyclophosphamide have been associated with the development of PCP; however, new immunomodulatory agents such as Tumor Necrosis Factor-*α* inhibitors and other monoclonal antibodies have also more recently been implicated.

Because PCP in the non-HIV population is more fulminant and has a higher mortality than in the HIV patients, this retrospective analysis, from a large tertiary medical center, was undertaken to address baseline conditions and outcome of PCP in HIV-negative patients and to identify groups at risk for PCP due to the use of corticosteroids or other immunosuppressant drugs.

## 2. Materials and Methods

### 2.1. Patient Population

Patients with PCP diagnosis were identified by a computerized search of the Mayo Clinic medical records, between 2006 and 2010. We excluded patients with HIV, unconfirmed PCP, or diagnosis established at another institution and those who were younger than 18 years of age. All individuals provided consent allowing the use of their records. Approval was obtained from the Mayo Clinic Rochester institutional review board (09-008573).

### 2.2. Diagnosis of PCP

PCP diagnosis required symptoms of pneumonia and positive microbiologic testing at our institution. Positive microbiologic testing was defined by either positive real-time polymerase chain reaction (PCR) assay or smear for* Pneumocystis jirovecii* performed on spontaneous sputum, induced sputum, bronchoalveolar lavage (BAL), or tissue samples. It is noteworthy that the nonnested PCR we use recognizes the single copy of the* PcCdc2* gene, providing added sensitivity for clinical diagnosis of PCP, as this technique has been specifically designed to detect clinical pneumonia and not colonization [8].

### 2.3. Data Analysis

Data are expressed as the mean and standard deviation for continuous normally distributed variables and as the median and range for non-Gaussian distributed data. Potential differences in variables were determined using *χ* ^2^ analyses and Fisher exact tests. Differences were considered significant if *P* values were < 0.05.

## 3. Results

### 3.1. Baseline Data and Underlying Conditions

**Figure 1:**
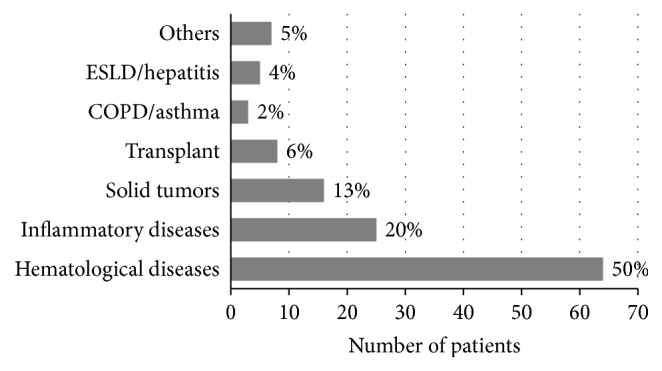
Underlying conditions in non-HIV patients with PCP (COPD: Chronic Obstructive Pulmonary Disease, ESLD: End Stage Liver Disease).

A total of 128 patients with PCP were identified during the study period. Eighty-six patients (67%) were male. The median age was 65.5 years (range, 19–89 years). Hematological malignancies were the most frequently found baseline condition (50%), followed by underlying inflammatory processes (20%) and solid tumors (13%) (Figure 1).

### 3.2. Prior Corticosteroid and Immunosuppressant Use

One hundred and fourteen (89%) patients received steroids or other immunosuppressive therapy prior to the diagnosis of PCP (Table 1). Corticosteroids were in the medication lists of ninety-eight patients (76%). Three were on corticosteroids for <4 weeks. Of those who received corticosteroids for >4 weeks, we identified three mayor groups: patients receiving daily corticosteroids, patients receiving corticosteroids intermittently during chemotherapy, and those on daily corticosteroids and as part of their chemotherapy regimens (Table 1). Sixteen patients (13%) were receiving other immunosuppressive therapy but had not received corticosteroids and 14 patients (11%) had not received corticosteroids or immunosuppressive therapy (Table 1). Rituximab was found in 23% of the patients receiving immunosuppressant drugs and methotrexate in 16.48%. Interestingly, 55 patients (43%) were not receiving daily doses of corticosteroids and of those 30 patients (23%) had not received corticosteroids at all in the period leading to PCP (Tables 1 and 2). For patients receiving only chronic systemic corticosteroids the mean prednisone equivalent dose was higher (27.73 mg/d) than if they were receiving corticosteroids plus other immunosuppressive agents (21.34 mg/d).

### 3.3. Use of PCP Prophylaxis

The use of PCP prophylaxis in non-HIV patients at risk for* Pneumocystis* pneumonia is not clearly defined. In our study, only 7 patients were receiving PCP prophylaxis, 3 were on pentamidine (300 mg nebulized monthly), 1 was on single strength trimethoprim 80 mg-sulfamethoxazole 400 mg (SS TMP-SMX) three times a week, 1 was on SS TMP-SMX after dialysis, 1 was on SS TMP-SMX daily, and 1 was on double strength, trimethoprim 160 mg-sulfamethoxazole 800 mg (DS TMP-SMX) twice a week. The baseline characteristics of these patients including their underlying condition and other medication used were outlined in Table 3. No association was found among the use of prophylaxis and underlying illness, specific immunosuppressant therapy, or steroid use.

### 3.4. Diagnosis of PCP

The laboratory diagnosis of PCP was made from the BAL in 81 patients (63%), sputum in 40 (31%), tracheal secretions in 3 (2%), and biopsy in 4 (3%).* P. jirovecii* was identified by nonnested real-time PCR of the single copy* Pccdc2 *gene in 93 (73%) cases, by examination of silver stained smear in 31 (24%), and by tissue examination with silver stain in 2 (2%).

### 3.5. Clinical Course

**Figure 2:**
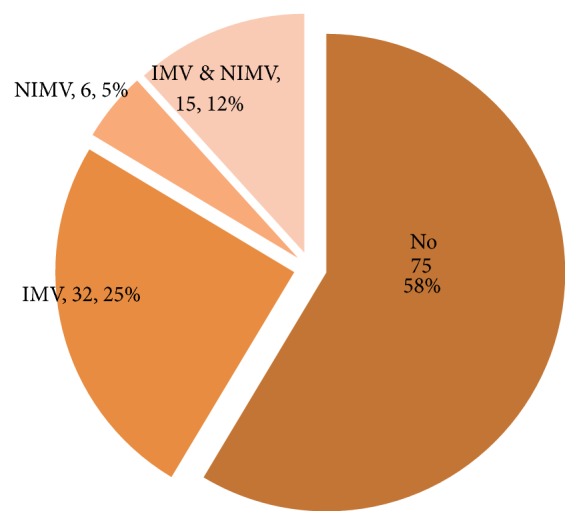
Requirement for mechanical ventilatory support (IMV: invasive mechanical ventilation, NIMV: noninvasive mechanical ventilation).

Eighty-three (65%) out of 128 of the patients had O_2_ saturation < 91% on admission. Data was not available in 4 patients. The mean hospital admission days were 15 (range, 0–124 days). Sixty-seven patients required admission to the Intensive Care Unit (ICU) and of those 53 individuals received mechanical ventilatory support (Figure 2). The mean ICU length of stay was 6.28 days (range, 2–52 days).

The median total lymphocyte count was 0.55 × 10^9^/L (range, 0.06–540 × 10*e* ^9^/L). The CD4 lymphocyte level was determined in only 23 patients and CD19 B-lymphocyte counts were determined in 10 patients (Table 4). In 4 patients the CD4 counts were found to be higher than 200/*μ*L; however, the CD19 B-cell count was less than 60/*μ*L in those patients.

In 67 (52%) patients, coinfection with other potential pathogens was observed. The most prevalent microorganisms present on laboratory testing are shown in Table 5. In one patient, active tuberculosis was also detected. The development of respiratory failure and the presence of coexisting infections showed a trend towards association but were not statistically significant (*P* = 0.073). No association was found with mortality, underlying disease, steroid use, or chemotherapy.

### 3.6. Treatment

One hundred and twelve (87.5%) patients were treated with TMP/SMX. Seven of them required switching the medication due to adverse effects. Atovaquone was the initial treatment for 10 patients. Additionally, atovaquone was the second therapeutic choice in 4 patients that did not tolerate TMP/SMX. Three patients were treated with clindamycin plus primaquine as the first therapy, but one had to be switched due to the development of methemoglobinemia. Pentamidine was used in three patients, but in two of them, pentamidine needed to be changed to another agent due to adverse effects. One patient was treated with three different medications. Corticosteroids were used as adjunctive therapy (CAT) in one hundred and four (81.25%) patients. Half of the patients (50%) treated with CAT required ICU level care. In contrast, only 2.34% of the patients who did not receive CAT were treated in the ICU (*χ* ^2^, *P* < 0.0001).

Seventy-one out of 94 patients discharged from the hospital received secondary prophylaxis. SS TMP/SMX one tablet daily was prescribed in 21 cases, DS TMP/SMX one tablet three times a week in 19 cases, DS TMP/SMX once daily in 17 cases, atovaquone suspension 750 mg twice a day in 5 cases, pentamidine 300 mg nebulized monthly in 5 cases, SS TMP/SMX 1 tablet twice a week in two cases, and dapsone 50 mg once daily in one case. Only two patients had a secondary episode of PCP, and both of them had been receiving secondary prophylaxis (SS TMP/SMX single strength daily in one case and DS TMP/SMX daily in the other case).

### 3.7. Mortality

**Figure 3:**
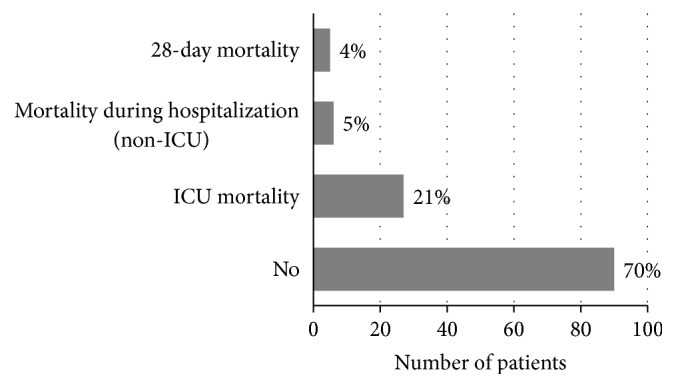
Mortality in non-HIV patients with PCP.

The highest mortality observed was in the group that required ICU with 27 out of 67 (40%) deaths during the ICU admission and a 28-day mortality of 49%. By comparison, the overall in-hospital mortality of those patients that did not require ICU was of 26% with a 28-day mortality 30% (Figure 3). The need of ventilatory support was related to mortality (*P* < 0.0001) but not the presence of coinfection with other pathogens (*P* = 0.16).

## 4. Discussion

In contrast to AIDS patients, the number of cases in the non-HIV population continues to increase with an average annual increase of 9% (*P* < 0.001) [9]. In this study, patients with hematological malignancies represented the largest portion of the group (50%) followed by inflammatory diseases and solid tumors (13%). Interestingly, 35 patients had also a diagnosis of COPD or emphysema, both preexisting lung diseases and potential contributors to the development of PCP [9].

As* Pneumocystis* cannot be cultured, the diagnosis of PCP has relied on the direct histological identification of the organisms. Recently, newer PCR techniques have offered improved means to diagnose PCP. While these techniques increase the diagnosis sensitivity they may detect colonization in addition to infection, particularly with nested PCR. Therefore, these highly sensitive techniques have to be applied in conjunction with compatible clinical and radiological data. At our institution, we have employed a real-time PCR that targets the* dcd2* gene, in which the detection threshold was set to avoid documenting colonization without infection [8]. Herein, our PCR was able to successfully diagnose patients not only from BAL but also from sputum. Sputum is easily obtained and can be used as an initial screening test, particularly when more invasive tests such as bronchoscopy are not readily available. A negative result, however, should not dissuade the provider to pursue further confirmatory testing when the suspicion for PCP is high.

Studies have suggested that PCP mortality rates vary according to the population at risk with cancer patients faring worse than other groups. The presence of respiratory failure is also an important independent predictive factor of mortality [7, 10]. In this study, 67 (52%) patients required ICU admission and 42% received mechanical ventilatory support. The ICU mortality was found to be 40% with a 28-day mortality of 49%. In the earlier study by Yale and Limper [11], 43% of their patients experienced respiratory failure with an in-hospital mortality of 66%. While both studies were derived from the same institution, the cohorts occurred in markedly different time periods and the observed differences in mortality can be likely attributed to improved ICU care and support particularly since the initiation of goal direct ICU care, lung protective ventilation strategies, increased PCP awareness with earlier empiric therapy, and perhaps changing selection criteria for ICU admission.

Studies comparing PCP among HIV-negative and HIV-positive patients have shown higher rates of intubation and mechanical ventilation in the non-HIV patients [12]. In Festic's series of HIV-negative patients with PCP and acute respiratory failure, there was an association of intubation delay with hospital mortality that persisted even after adjusting for the severity of illness (*P* = 0.03) [13]. In our study, the mortality of patients that required mechanical ventilation was significantly higher than those that did not. While in HIV-negative patients the initial antimicrobial treatment failure for PCP was an independent predictor of poor clinical outcome [14], in our study the primary reason for alternative treatment was medication intolerance and adverse effects, rather than failure to respond to the primary treatment which was usually TMP-SMX followed by atovaquone.

CAT is a practice well established in HIV-infected patients, particularly if they have associated hypoxemia [15]. The data supporting CAT for HIV-negative patients is less definitive [16, 17]. However, the ATS Fungal Treatment Guidelines recommend its use in moderate to severe PCP, irrespective of the underlying immunosuppressive condition [18]. Herein, up to 80% of the patients received CAT; of those 30% of patients still expired within 28 days of the diagnosis. Unfortunately, the retrospective nature of our study does not allow us to directly answer the question of whether corticosteroids were beneficial to this population, and our observations likely also reflect the mortality related to the severity of the underlying disease. It should be noted that our practice is most likely to use CAT for those with moderately severe to severe PCP, particularly if there is respiratory failure.

Corticosteroids have been long recognized as major risk factor for the development of PCP. The study by Yale and Limper reported that daily prednisone dose of 16 mg a day over a median of 8 weeks increased the risk of developing PCP [11]. One of the main findings in our study is that up to 45% of the patients either had not received corticosteroids in the 4-week period prior to the development of PCP or had not been taking daily corticosteroids prior to the infection. Of those, 13% had been receiving other immunosuppressive therapies and 11% had not received any immunosuppressive drugs. These data strongly suggest that while daily corticosteroids are an important risk factor for the development of PCP, patients on high intermittent steroids, chemotherapy, and severe immunosuppressive diseases even in the absence of treatment are also at a higher risk. Nineteen patients on intermittent corticosteroids as part of their chemotherapy regimen were receiving the equivalent to 70 mg of prednisone a day. Hence, we believe that high intermittent corticosteroid therapy is also an important risk factor for developing PCP.

Rituximab, MTX, and everolimus were the most frequent immunosuppressive agents used either alone or in combination with other immunosuppressive medications in patients that did not receive corticosteroids. The association of rituximab and PCP has been already reported by our group and several of these patients were included in that prior series [19]. The data suggested that B-cells play an important role in the host immune defense against PCP. Here, we observed that a number of our patients developed PCP with CD4 counts >200 cells/*μ*L. Interestingly, some of these patients had low B-cells numbers (<60 cells/*μ*L) supporting the contention that B-cell/T-cell interactions may contribute to overall PCP defense. Observations in mice models and human “in vitro” studies revealed that B-cells not only are responsible for antibody production to* Pneumocystis* but also act as potent modulators of CD4+ T-cell and neutrophil responses [20–23]. While we admit that the numbers of such patients in our cohort are very small, the role of B-cell related activity in PCP prevention remains an important area for continued investigation.

Therefore, while monitoring CD4 counts may add value in predicting PCP risk, this practice alone appears insufficient as the sole means to predict infection risk across immunosuppressed patients without HIV [24]. While many experts suggest discontinuing prophylaxis when CD4 cells are >200/*μ*L for at least 6 months [25], we believe that this practice should be applied carefully and in an individualized manner. Furthermore, based on our data, particular attention should also be directed to patients on intermittent high doses of corticosteroids and those that are felt to be severely immunosuppressed due to their underlying systemic condition.

The main limitation of this study is the retrospective nature of our analysis. But overall, the characteristics of our patients were similar to those described in other HIV-negative adults series from Europe and Israel [7, 24, 26, 27]. Our study reflects that most cases occurred in patients that did not receive adequate chemoprophylaxis and emphasized the lack of consensus as when to implement it. Hence, while the decision of chemoprophylaxis must be individualized we must keep in mind that PCP results in high associated mortality. Furthermore, this report emphasizes the need for continuing high levels of suspicion by care providers of immunosuppressed patients even if they are on intermittent corticosteroids.

In summary, our study demonstrates that PCP remains a serious complication for the immunosuppressed population without HIV infection and calls for continuing awareness and appropriate chemoprophylaxis if significant immunosuppression of these patients is anticipated.

## Figures and Tables

**Table 1:** Prior corticosteroid and immunosuppressant use in non-HIV patients with PCP.

	Steroid use						
	Count total %	No	Daily steroids	Steroids^*∗*^ with chemotherapy	Daily steroids and with chemotherapy^#^	Steroids burst <4 weeks	
Immunosuppressant use	No	14 10.94	20 15.63	0 0.00	0 0.00	3 2.34	37 28.91
	Yes	16 12.50	36 29.69	25 19.53	14 10.94	0 0.00	91 71.09

		30 23.44	56 43.75	25 19.53	14 10.94	3 2.34	128

^*∗*^Steroids intermittently with chemotherapy.

^#^Steroids daily and intermittently with chemotherapy.

**Table 2:** Daily corticosteroids use in non-HIV patients with PCP.

	Count total %	No steroid use	Only steroids	Steroids and chemotherapy	Steroids burst <4 weeks	
Steroid use	Intermittent use	30 23.44	0	25 19.53	0	55 42.97
	Daily use	0	56 43.75	14 10.94	3 2.34	73 57.03

		30 23.44	56 43.75	39 30.47	3 2.34	128

**Table 3:** Characteristics of patients that received PCP prophylaxis.

Gender	Age (years)	Prophylaxis used	Baseline disease	Steroid use^*∗*^	Other immunosuppressants
Male	20	Inhaled pentamidine	Hematological malignancy	No	Tacrolimus

Male	61	Inhaled pentamidine	Hematological malignancy	Only with chemotherapy	Methotrexate, mercaptopurina, and vincristine

Female	68	Inhaled pentamidine	Hematological malignancy	Only with chemotherapy	ABVD (doxorubicin, bleomycin, vinblastine, and dacarbazine)

Male	72	TMP-SMX (SS) 3x per week	Transplant (Renal and liver)	Daily (5 mg a day)	Tacrolimus and mycophenolate mofetil

Male	46	TMP-SMX (DS) 2x per week	Inflammatory	Daily (30 mg a day)	Azathioprine

Male	29	TMP-SMX (SS) Once a day	Transplant (Lung)	Daily (10 mg a day)	Cyclosporine and azathioprine

Female	27	TMP-SMX (SS) After dialysis	Inflammatory	Daily (10 mg a day)	No

^*∗*^Prednisone equivalent.

**Table 4:** Laboratory data in non-HIV patients with PCP.

*Total number of patients*	*n* = 128

*CD4 (number of patients)*	*n* = 23
Maximum	1221
Mean (std. dev.^a^)	221.39 (305.73)
Median	10

*CD4 (without outlier)*	*n* = 22
Maximum	984
Mean (std. dev.^a^)	175.95 (219.50)
Median	96

*CD19-B cells (number of patients)*	*n* = 10
Maximum	135
Mean (std. dev.^a^)	29.2 (44.77)
Median	6.5

*CD4/CD19-B cells (number of patients)*	*n* = 10
	33/135
	18/0
	146/70
	15/0
	394/3
	87/14
	1221/1
	383/4
	100/9
	387/56

^a^Std. dev.: standard deviation.

**Table 5:** Potential coinfecting organisms in non-HIV patients with PCP.

More prevalent organisms	
*Candida *sp.	23
*Cytomegalovirus*	11
*Aspergillus *sp.	7
*Herpes simplex type I*	4
*Staphylococcus aureus*	6
*Coagulase negative Staphylococcus *	4
*Pseudomonas aeruginosa*	3
*Klebsiella pneumoniae*	3
*Penicillium *sp.	3

## Abbreviations

AIDS: Acquired immunodeficiency syndrome
BAL: Bronchoalveolar lavage
CAT: Corticosteroids adjunctive therapy
COPD: Chronic Obstructive Pulmonary Disease
HIV: Human Immunodeficiency Virus
ICU: Intensive Care Unit
IS: Immunosuppressive
PCP: * Pneumocystis* pneumonia
PCR: Polymerase chain reaction
TMP-SMX: Trimethoprim-sulfamethoxazole.
